# miRNA-576-5p promotes endometrial cancer cell growth and metastasis by targeting ZBTB4

**DOI:** 10.1007/s12094-022-02976-8

**Published:** 2022-12-20

**Authors:** Chen Chen, Qing Zhang, Beihua Kong

**Affiliations:** 1 Department of Obstetrics and Gynecology, Qilu Hospital, Cheeloo College of Medicine, Shandong University, 107 Wenhua Xi Road, Jinan, 250012 Shandong People’s Republic of China; 2 Department of Gynecology, The Second Hospital, Cheeloo College of Medicine, Shandong University, 247 Beiyuan Street, Jinan, 250033 Shandong People’s Republic of China; 3 Key Laboratory of Gynecologic Oncology of Shandong Province, 107 Wenhua Xi Road, Jinan, 250012 Shandong People’s Republic of China

**Keywords:** miR-576-5p, Endometrial cancer, ZBTB4, Proliferation, Metastasis

## Abstract

**Purpose:**

MicroRNAs (miRNAs) have already been shown to have a strong correlation with the invasion and metastasis capacity of tumor cells. The present research examined the function of miRNA-576-5p (miR-576-5p) in the development of endometrial cancer (EC).

**Methods:**

miR-576-5p and ZBTB4 expression in EC and benign endometrial tissues was measured using quantitative real-time PCR (qRT-PCR) and western blot. To evaluate the proliferation ability of tumor cells in vitro, 2,5-diphenyl-2H-tetrazolium bromide (MTT) and colony formation assays were carried out. The effect of miR-576-5p on the proliferation ability of EC cells in vivo was measured by the tumor formation in nude mice. The migration and invasion ability of tumor cells was determined using the transwell assay. To confirm the association between expressions of miR-576-5p and zinc finger and BTB domain containing four (ZBTB4), western blot, qRT-PCR, and luciferase assay were carried out.

**Results:**

miR-576-5p expression increased significantly in EC samples than in benign endometrial tissues. The level of miR-576-5p was significantly higher in the polymerase ε (POLE) ultramutated subgroup compared to the other three subgroups. High levels of miR-576-5p expression were linked to a shorter progression-free interval time in the copy number high subgroup. Furthermore, upregulated miR-576-5p facilitated EC cell invasion and migration in vitro and promoted the proliferation of EC tumor cell lines both in vitro and in vivo. Moreover, this study showed that the expression of *ZBTB4* decreased in patients with EC, and the dual-luciferase reporter assay confirmed that miR-576-5p binds directly to the 3′-UTR of *ZBTB4* and inhibits the expression of *ZBTB4*. An increase in miR-576-5p expression leads to a decrease in the mRNA and protein expression level of *ZBTB4*. The effects of miR-576-5p can be reversed by overexpression of *ZBTB4*.

**Conclusion:**

miR-576-5p promoted proliferation and metastasis capacity of EC cells by inhibiting *ZBTB4 expression*. We hypothesized that miR-576-5p could be a prospective therapeutic target for EC.

**Supplementary Information:**

The online version contains supplementary material available at 10.1007/s12094-022-02976-8.

## Introduction

In the United States and Europe, endometrial cancer (EC) ranks high among cancers of the female reproductive system [1, 2]. The National Comprehensive Cancer Network (NCCN) guidelines recommend staging surgery, chemotherapy, and radiation therapy as preferred treatments for EC. Although most patients receive an early diagnosis and have a good prognosis after standard surgical treatment, EC remains the only gynecologic malignancy with an increasing mortality rate [3, 4]. Therefore, it is essential to elucidate the key molecular markers of pathogenesis, metastasis, prognosis, and chemoresistance among patients with this disease. In 2013, the Cancer Genome Atlas (TCGA) categorized EC into the polymerase ε (POLE) ultramutated, microsatellite instability hypermutated, copy number-low, and copy number high subgroups [5]. This classification of EC demonstrates the relationship of this disease with patient survival and provides valuable information for clinical prognosis and risk prediction.

MicroRNAs (miRNAs) are a family of small non-coding RNAs (ncRNAs) of 19–24 nucleotides in length. Most miRNAs are located within intronic or exonic regions and are processed by RNA Polymerase II/III to form pri-miRNAs, then by Drosha and Pasha [also known as DiGeorge syndrome critical region 8 (DGCR8)] to generate pre-miRNAs, and at last by cytoplasmic ribonuclease (Dicer/TRB) to form mature miRNAs. By binding to the 3′-untranslated regions (UTR) of the target gene, miRNAs control the expression of certain genes, which in turn inhibits translation or destabilizes the target mRNA [6, 7]. MiRNAs have fundamental roles in cell differentiation, organism development and metabolism, and tumorigenesis [8]. The widespread involvement of miRNA in human diseases emphasizes their diagnostic and therapeutic potential [9]. Many efforts have been devoted to targeting endogenous miRNAs to achieve therapeutic effects. In recent years, investigators confirmed hundreds of miRNAs, such as let-7b, miRNA-34 family, miRNA-182, and miRNA-652, to be crucial in the pathogenesis of EC [10–12]. Furthermore, researchers discovered that microRNA-576-5p (miR-576-5p) plays a role in the progression of many cancers, such as gastric cancer, esophageal squamous cell, colorectal cancer, breast cancer, and melanoma [13–19], but its role in EC has not been elucidated so far.

A known repressor of transcription, zinc finger and BTB domain containing 4 (ZBTB4), suppresses the pathogenesis of many malignant tumors, such as prostate cancer [20], breast cancer [21], lung cancer [22], and Ewing sarcoma [23]. Furthermore, overexpression of ZBTB4 is associated with prolonged relapse-free survival of patients with cancers above [20–23]. Furthermore, ZBTB4 has been linked to the development of non-neoplastic diseases such as HIV and Alzheimer’s disease [24–26].

In this study, the levels of miR-576-5p in EC patients, as well as the role of this miRNA in EC pathogenesis, were determined. We found that miR-576-5p could promote the malignancy of EC cells, suggesting that miR-576-5p and its target gene, *ZBTB4*, could be used as targets for EC treatment.

## Materials and methods

### Tissue sample collection

In this study, 38 patients with EC underwent surgical staging at Qilu Hospital, Shandong University, from December 2014 to May 2018 (EC group), and 16 patients who underwent hysterectomy because of leiomyomas from March 2018 to May 2018 (benign group) were included. Endometrial samples were collected from both groups, and tumors in the EC group were staged and graded following the criteria presented by the International Federation of Gynecology and Obstetrics (FIGO) 2009. Before staging surgeries, no patients have been treated with progesterone drugs, chemotherapy, or radiation therapy. The samples were obtained from the patients after resection and stored in a − 80 °C refrigerator. Benign or malignant natures of all samples were confirmed using frozen pathology examination before RNA or protein extraction. Each patient provided consent in a written format before the operation. Furthermore, the experiment followed the guidelines set by the Ethics Committee of Qilu Hospital, Shandong University.

### Cell culture and cell lines

The human EC cell lines (i.e., AN3-CA and Ishikawa cells) were obtained from the American Type Culture Collection (ATCC) (Manassas, VA). The Chinese Academy of Science (Shanghai, China) provided the human embryonic kidney 293 T (HEK-293 T) cell line. The AN3-CA cell line was cultivated in Modified Eagle’s Medium (MEM) supplemented with 10% fetal bovine serum (FBS), 100 mM pyruvic acid, and 100 mM non-essential amino acids. The Ishikawa and HEK-293 T cell lines were cultured in DMEM (Dulbecco’s Modified Eagle’s Medium) containing 10% FBS. The aforementioned cultural media were all purchased from Gibco (Grand Island, USA). All the cell lines were cultured under a humidified incubator with 5% CO_2_ at 37 °C.

### RNA extraction and quantitative real-time polymerase chain reaction (qRT-PCR)

TRIzol reagent (Invitrogen) was used to isolate total RNA. The RNA in the samples was quantified using spectrophotometry. The extracted RNA is of good quality if its optical density (OD) 260/280 is between 1.8 and 2.0. To synthesize miRNA’s cDNA and reverse transcribe the mRNA, the one-step Primescript miRNA cDNA Synthesis Kit and the Prime Script RT Reagent Kit were used, respectively (both kits were purchased from Takara Bio). qRT-PCR was performed with StepOne Plus Real-Time PCR System (QuantStudio3; Thermo Fisher Scientific, USA). Sequences of primers used in this study are listed in Supplementary Table 1. U6/ACTB was used as the endogenous control for miRNAs/mRNAs. The 2^−∆∆Cq^ method was used to determine the relative expressions of specific genes.

### Transient and stable transfection

The overexpression and knockdown of miR-576-5p were achieved using mimics and inhibitors. The inhibitor, mimics, and respective negative control (NC) were obtained from GenePharma (Shanghai, China). Small interfering RNAs (siRNAs) targeting *ZBTB4* and NC were synthesized by GenePharma. The ZBTB4 overexpressing vector in pEnter and corresponding NC were synthesized by Vigenebio (Shandong, China). Transient transfection of cells was achieved with lipofectamine 2000 (Invitrogen). The lentiviruses expressing pre-miR-576-5p and corresponding NC were synthesized by Genechem (Shanghai, China). Prior to stable transfection, 1 × 10^5^ cells were seeded in each well of a six-well plate containing antibiotic-free medium. Twenty-four hours (h) after cell seeding, the lentiviruses were introduced to the culture medium following the manufacturer’s instructions. The medium was replaced with fresh medium comprising 2 μg/mL puromycin (Sigma-Aldrich, Missouri, USA) 24 h later, and this procedure was repeated every day for 2 weeks to obtain stably transfected cells. The siRNAs and NC sequences used in this study are listed in Supplementary Table 2.

### Cell migration and invasion assay

Twenty-four hours after siRNA or NC transfection, the cells were resuspended in the FBS-free medium. Then, 1.8 × 10^5^ treated cells in 200 μL serum-free medium were inoculated into the upper chamber of Transwell chambers (BD Biosciences, USA) equipped with or without Matrigel (40 μL 8 mg/mL stock solution) for invasion or migration capacity testing. A standard culture medium was placed in the lower chamber. The cells were cultivated for a specific duration at 37 °C. The membranes were washed with PBS, fixed with methanol, and then stained with 0.5% crystal violet. Cells adhered to the submembrane layer were counted under a microscope to determine their migration and invasion capacity.

### Cell proliferation assay

Twenty-four hours after siRNA or NC transfection, 1000 transfected cells in 100 μL of medium were transferred into each well of 96-well plates and incubated until the cells adhered. Cell proliferation was measured every 24 h for a total of 5–7 days. Then, 20 μL of 5 mg/mL MTT (Sigma-Aldrich; Missouri, USA) was added into each well at a fixed time and the cells were incubated for another 4 h. Next, 100 µL DMSO (Sangon Biotech, Shanghai, China) was added to each well after removing the supernatant carefully. Subsequently, the absorbance value at 490 nm was detected by a Varioskan Flash microplate reader (Thermo Fisher Scientific, USA). All the experiments were performed in triplicate.

### Cell cycle and apoptosis analysis

The treated cells were trypsin digested, centrifuged, washed with PBS, and fixed overnight at 4 °C using 70% ice-cold ethanol. The next day, cells were rinsed using PBS, and then stained with propidium iodide (PI) (BD Biosciences, New Jersey, USA). The PI signal was detected using a FACS Calibur Flow Cytometer (FC500, Beckman Coulter, USA). The Annexin V FITC kit (Invitrogen) was used to test the apoptosis. All the assays were carried out following the manufacturer’s instructions.

### Colony-formation assay

A total of 500 treated cells were transferred into each well of a 6-well plate, and cultured for 10–20 days. After washing with PBS, cells were fixed with 100% methanol and stained with 0.5% crystalline violet. The experiment was carried out in triplicate and only clones with more than 50 cells were counted.

### Western blot

The transfected cells or ground endometrial tissue were mixed with RIPA lysis buffer containing 100 µM NaF and 1% PMSF (all from Beyotime, China). After incubation on ice for 30 min, the mixture was centrifuged and the supernatant was collected. Protein concentration was measured using the BCA assay (Thermo Scientific, USA). The protein samples were subsequently processed in the following steps: the samples were loaded into sodium dodecyl-sulfate polyacrylamide gel electrophoresis (SDS-PAGE) gel; the proteins were transferred to PVDF membranes (with a thickness of 0.2 µm) (Millipore, Billerica, MA, USA); the membranes were blocked with 5% skimmed milk for at least one hour; the membranes were incubated with primary antibodies at 4 °C overnight; the membrane was incubated with secondary antibodies for 1 h. ECL Plus detection reagent (PerkinElmer, USA) was used to visualize the bands using the ImageQuant LAS 4000 system (GE Healthcare Life Sciences, MA, USA). The anti-β-actin antibody (CST, No.3700, 1:1000 dilution) was used as a loading control. Antibodies used in the current study included anti-ZBTB4 (Abcam, ab106554), anti-Sp1 (Abcam, ab227383), and anti-VEGF (Abcam, ab32152) antibodies. Antibodies for EMT and cell cycle detection included anti-ZEB-1 (CST, No.3396), anti-*N*-cadherin (CST, No.13316), anti-E-cadherin (CST, No.3195), anti-Vimentin (CST, No.5741), anti-beta-catenin (Abcam, ab16051), anti-survivin (CST, No.2808), and anti-CCND1 (Abcam, ab16663). All these antibodies were diluted at 1:1000 except for anti-beta-catenin and anti-CCDN1 antibodies, which were at 1:4000 and 1:500 respectively.

### Target prediction and dual-luciferase reporter assay

Web-based miRNA databases, such as miRDB (http://www.mirdb.org/), TargetScan (http://www.targetscan.org/), and MiRanda (http://www.microrna.org), were searched to identify potential target genes of miR-576-5p. Furthermore, homo sapiens miR-576-5p was predicted to bind to the 3′-UTR of ZBTB4. The interaction of miR-576-5p with 3′-UTR of ZBTB4 was validated using a luciferase assay. The reporter luciferase constructs containing the 3′-UTR of ZBTB4 and the corresponding mutant sequence were purchased from Vigenebio (Shandong, China). HEK-293 T cells (2–3 × 10^4^/well) were seeded in wells of a 96-well plate and co-transfected with 50 ng MT or WT ZBTB4′ 3′-UTR vector and 0.5 pmol miR-576-5p mimic or NC, using lipofectamine 2000 reagent. Twenty-four hours after transfection, the luciferase activity was determined using a Dual-Glo Luciferase Assay (Promega, Wisconsin, USA). The ratio of activities between luciferase obtained from firefly and renilla was used to normalize the firefly activity, which was used as control.

### Xenograft tumor formation assay

After digestion and resuspension, the concentration of stably transfected tumor cells was adjusted to 1.0 × 10^7^/ml. Then, 200 µL of suspension was injected into the axilla on both sides of the same female BALB/c nude mouse (aged 4–5 weeks). All the mice used in this study were purchased from the Nanjing Biomedical Research Institute at Nanjing University. After 4 weeks, the mice were euthanized and the tumors were collected, weighed, and photographed. The selected tissues were examined by hematoxylin and eosin (H and E) staining and immunohistochemistry (IHC) analysis. All experiments related to mice were carried out following the guidelines for the Care and Use of Laboratory Animals of China.

### Immunohistochemistry (IHC) assay

Sections of paraffin-embedded EC and benign endometrial tissues (4 μm-thick) were deparaffinized, hydrated, hydrogen peroxide treated, and boiled for antigen repair. The sections were incubated with the anti-ZBTB4 antibody (1:200 dilution) (Abcam, ab106554) overnight at 4 °C in a humidified container. For staining and counterstaining, the DAB chromogenic kit (ZSGB Bio, China; stained brown) and Gill’s hematoxylin (Solarbio Bio, China) were used, respectively. ZBTB4 expression in tissues was indicated by the distribution and intensity of positive staining.

### Statistical analysis

The data obtained in this study were evaluated by SPSS software (version 18.0) (SPSS Inc., Chicago, USA). The unpaired Student’s *t* test and *χ*^*2*^ test were used to assess correlations and significant between-group differences. The Kaplan–Meier method and log-rank test were used to calculate overall survival. Experimental data were presented as mean ± standard error (SEM), and statistical significance was defined as *P* < 0.05.

## Results

### miRNA-576 expression is elevated in endometrial cancer

**Fig. 1:**
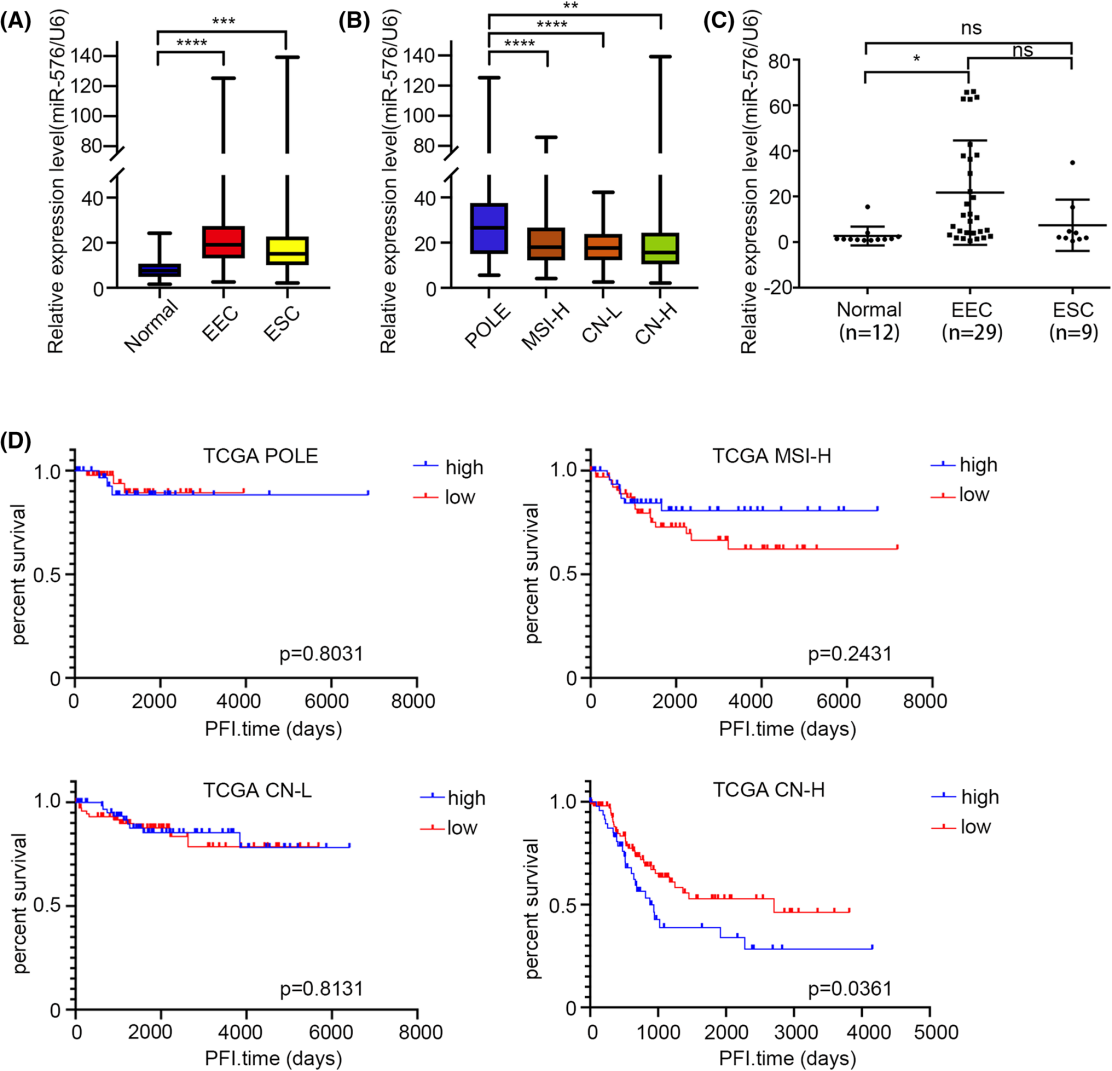
MicroRNA-576-5p is highly expressed in endometrial cancer and correlates with clinical information. **A** TCGA differential analysis identifies that microRNA-576 is upregulated in EEC and ESC patients than in normal endometrium samples. **B** And the level of miRNA-576 in POLE subgroups is higher than that in the other three subgroups. **C** miR-576 expression was detected in 38 EC tissues (29 EEC tissues and nine ESC tissues) and 12 normal endometrium samples by qRT-PCR. The expression level of miR-576 in EC samples was higher than that in normal endometrium tissues. **D** The high expression level of miR-576 exhibited shorter progression-free interval time than patients with low miR-576 expression in the copy number high subgroup. **p < 0.01, ***p < 0.001, ****p < 0.0001. *ESC* endometrial serous carcinoma, *POLE* polymerase ε (POLE) ultramutated subgroup, *MSI-H* microsatellite instable hypermutated subgroup, *CN-L* copy number-low subgroup, *CN-H *copy number high subgroup, *PFI* progression-free interval

Analysis of the miRNA-seq data from TCGA showed that the expression of miR-576-5p was increased in EC patients (Fig. 1A), especially in the POLE-ultramutated subgroup (Fig. 1B). qRT-PCR analysis revealed that the miR-576-5p expression level was significantly higher in EC tissues than in the benign endometrial tissues (Fig. 1C). We further analyzed the correlation between the expression level of miR-576 and clinicopathological parameters. Based on the average miR-576-5p expression levels, EC patients in the TCGA were separated into two groups. We found that the high level of the miR-576-5p group had a significantly shorter progression-free interval time than the low-miR-576-5p group in the copy number- high subgroup (Fig. 1D). Moreover, high levels of miR-576-5p were related to an early age of EC onset (Table 1).

**Table 1:** Association between miRNA-576 expression with clinicopathological parameters in patients with EC

Clinicopathological variable	*N*	miRNA-576 expression		*P*
		High	Low	
Age				
< 50	46	27	19	0.001*
≥ 50	469	163	306	
BMI				
< 28	145	47	98	0.695
≥ 28	321	110	211	
Menopausal				
Pre	51	22	29	0.215
Post	422	145	277	
Grade				
G1	97	29	68	0.152
G2	117	50	67	
G3	304	111	193	
Stage				
I–II	371	135	236	0.827
III–IV	147	55	92	
Lymph nodes				
Positive	56	20	36	0.977
Negative	323	116	207	
Recurrence				
Yes	61	29	32	0.061
No	457	161	296	

*P < 0.05； **P < 0.01

### Upregulation of miR-576-5p promotes the proliferation and growth capacity of EC cells in vitro and in a xenograft tumor model

**Fig. 2:**
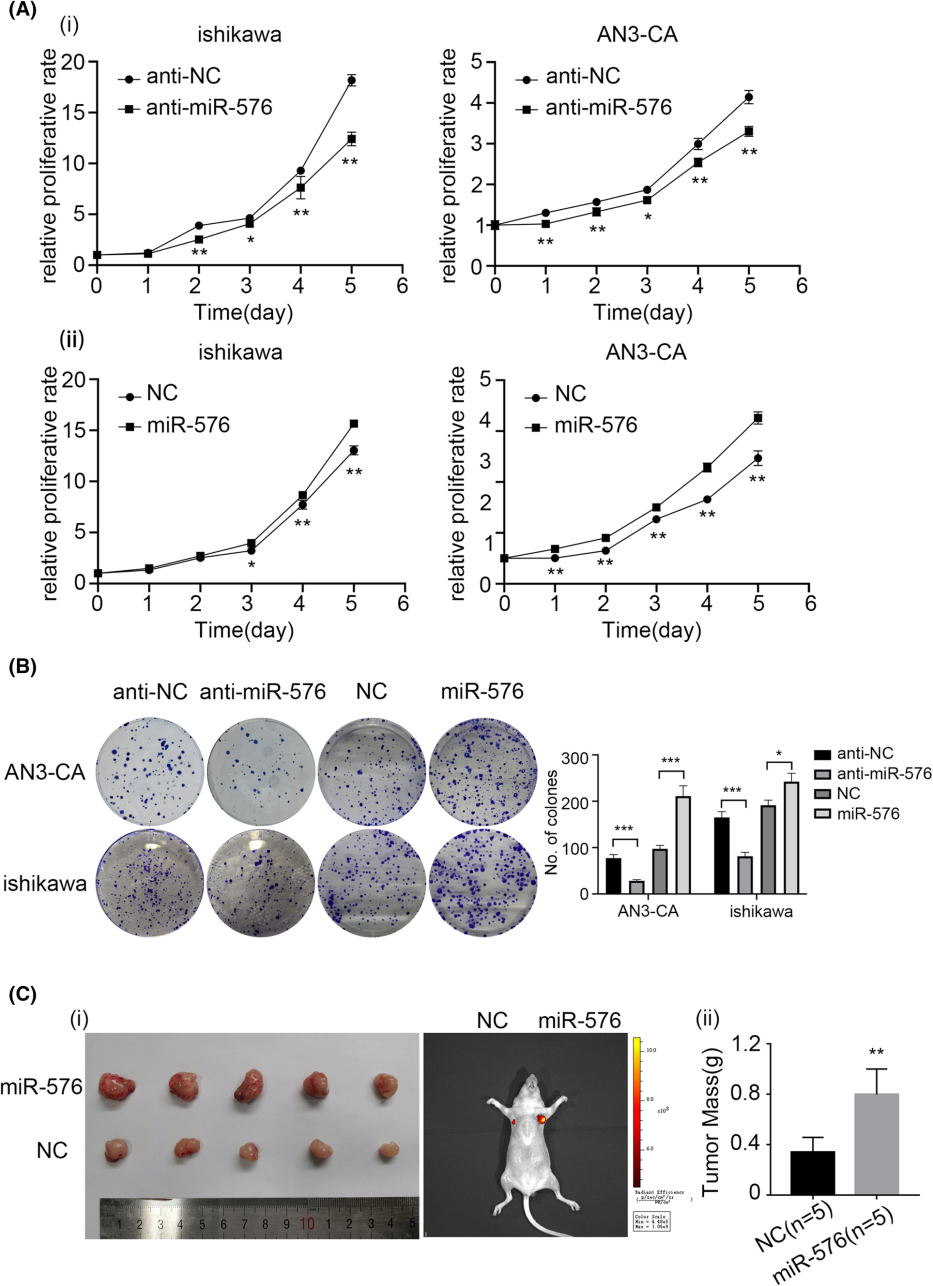
miR-576 promoted EC cells’ proliferation in vitro and in vivo. **A** Downregulation of miR-576 expression suppresses the proliferation of ishikawa and AN3-CA cells. Upregulation of miR-576 promotes the proliferation of EC cells. **B** Downregulation of miR-576 expression suppresses the colony formation of EC cells. Upregulation of miR-576 expression plays the opposite role. **C** The effect of miR-576 overexpression on tumorigenesis in vivo. (i) Overexpression of miR-576 promoted EC cell-derived tumor growth in a xenograft model. (ii) The tumor weight of overexpressed miR-576 was increased than that of NC

The biological function of miR-576-5p in EC cell lines was investigated. AN3-CA and Ishikawa cell lines were transiently transfected with miRNA-576 inhibitor/inhibitor-NC and miR-576-5p mimics/mimic-NC. miR-576-5p overexpression could significantly promote proliferation in the AN3-CA and Ishikawa cells. In contrast, downregulation of miR-576-5p inhibited the proliferative ability of EC cells, as indicated by the cell proliferation and clone formation assays (Fig. 2A, B). The Ishikawa cells stably transfected with pre-miR-576-5p or an NC vector were used to establish an EC mouse model. As shown in Fig. 2C, Ishikawa cells that overexpressed miR-576-5p had a significantly higher tumor weight than the NC group. Taken together, miR-576-5p can promote EC cell proliferation in vitro and in vivo.

### miR-576-5p overexpression promotes the invasion and migration capacity of EC cells

**Fig. 3:**
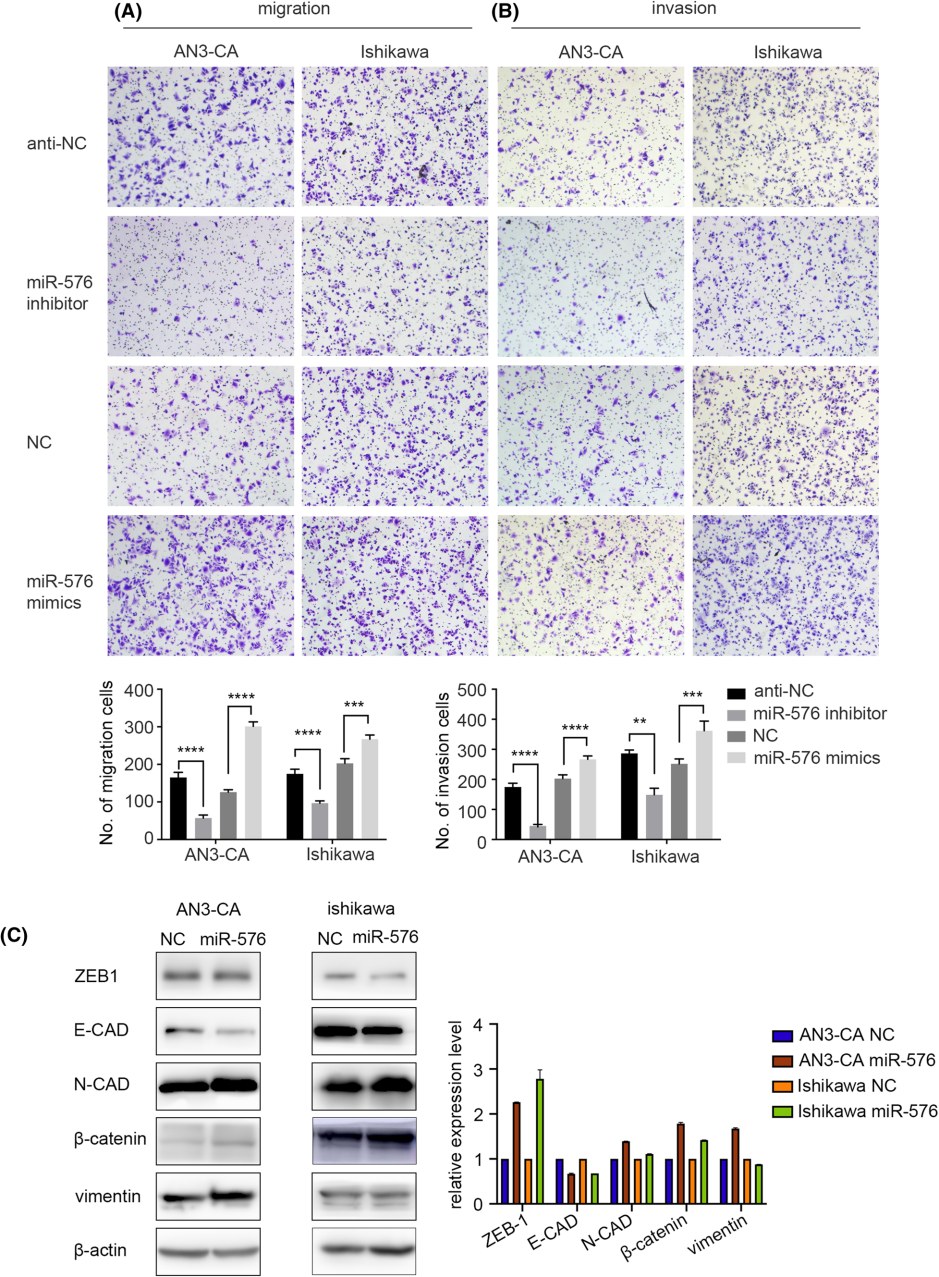
MiR-576 enhanced the migration and invasion ability of EC cells. **A** Inhibition of miR-576 suppressed the migration and invasion in Ishikawa and AN3-CA cells. **B** Overexpressed miR-576 enhanced both Ishikawa and AN3-CA cells’ ability to migrate and invade. **C** Western blots assay was used to detect the expression of the indicated mesenchymal markers. β-Actin was used as an internal control. *p < 0.05, **p < 0.01

The transwell assay was used to investigate the influence of miR-576-5p expression on the migration and invasion capacity of EC cell lines. We showed that downregulation of miR-576-5p reduced the migration and invasion of the cell lines; on the contrary, overexpression of miR-576-5p promoted the migration and invasion capacity of the EC cell lines (Fig. 3A, B). According to these findings, we concluded that upregulation of miR-576-5p expression could promote endometrial tumor metastasis in vitro. Furthermore, epithelial-mesenchymal transition (EMT) markers were used to investigate the molecular mechanism. We found that in EC cells, miR-576-5p overexpression upregulated several mesenchymal markers (ZEB-1, N-cadherin, vimentin, and β-catenin) and downregulated some epithelial markers (E-cadherin) (Fig. 3C). These data demonstrated that miR-576-5p could facilitate metastasis of EC cells.

### ZBTB4 is a target of miR-576-5p and regulated by miR-576-5p in EC cells

**Fig. 4:**
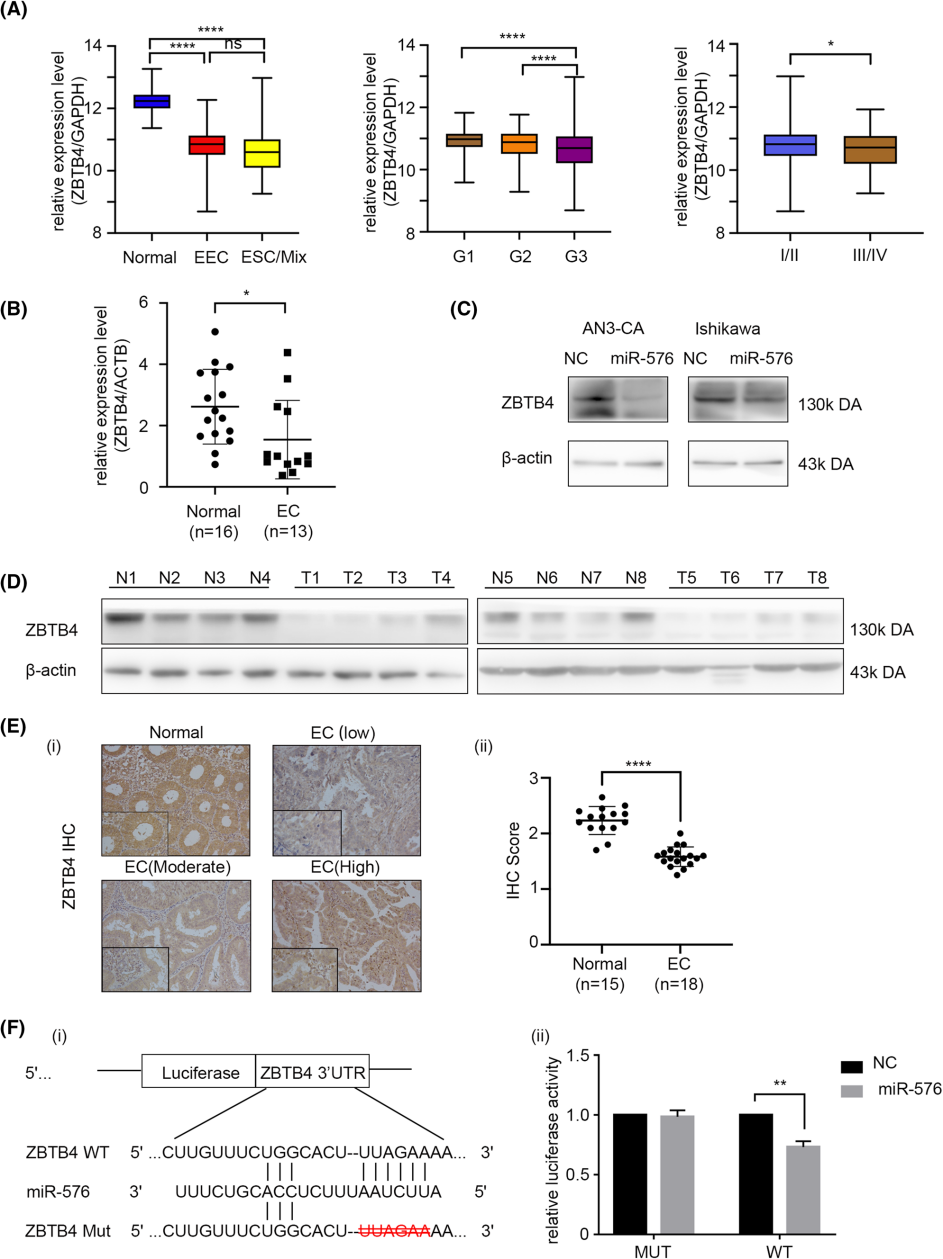
ZBTB4 is a target of miR-576. **A** In the TCGA database, the expression of ZBTB4 is downregulated in endometrial cancer, and the expression level corresponds with grade and stage. **B** ZBTB4 is downregulated in EC tissues than in benign endometrium tissues at the mRNA level. **C** The expression of ZBTB4 in Ishikawa and AN3-CA is negatively correlated with the miR-576 expression level. Upregulation of miR-576 can inhibit the expression of ZBTB4 in Ishikawa and AN3-CA cell lines. **D** The western blot assay confirmed that the protein level of ZBTB4 was higher in normal endothelial tissues than in cancerous tissues (N1-N8 are normal endometrial tissues; T1-T8 are endometrial cancer tissues). **E** Immunohistochemical analysis of ZBTB4 expression. (i) Representative IHC images of ZBTB4 in endometrial cancer tissue and benign endometrial tissue (ii) ZBTB4 expression in endometrial cancer tissues was lower than that in normal endometrial tissues. **F** ZBTB4 is the target gene of miR-576. (i) The binding sequence of miR-576 in ZBTB4 3′UTR. The deletion of the binding sequence of miR-576 in the ZBTB4 3′UTR region is a mutant type (MT). (ii) HEK 293 T cell line was transfected with negative control or miR-576 mimics and wild-type (WT) or mutant constructs containing ZBTB4 3′-UTR inserts linked to the luciferase gene, and after 48 h, luciferase activity was determined as described in the Materials and Methods. *p < 0.05, **p < 0.01, ***p < 0.001.ESC = endometrial serous carcinoma, I/II FIGO stage I/II, III/IV FIGO stage III/IV

To further investigate the molecular mechanism associated with miR-576-5p’s effects on EC cells, we performed a database search to identify targets of miR-576-5p and identified *ZBTB4* as a candidate target of miR-576-5p. RNAseq data downloaded from the TCGA Uterine Corpus Endometrial Carcinoma (UCEC) dataset demonstrated that *ZBTB4* expression was much higher in benign endometrial tissues than in EC tissues. Its level was related to the tumor grade and stage (Fig. 4A). These observations were verified using samples obtained from Qilu Hospital, both at the RNA and protein levels (Fig. 4B, D, E). To clarify whether *ZBTB4* is regulated by miR-576-5p, we further analyzed the relationship between *ZBTB4* and miR-576-5p mRNA levels; we found that they were negatively correlated (Supplementary Fig. 3B), which is consistent with what has been observed in the TCGA data (Supplementary Fig. 3C). Moreover, we examined the RNA and protein expressions of *ZBTB4* in EC cell lines that overexpressed miR-576-5p. We found that miR-576-5p upregulation could decrease the expression of *ZBTB4* at both the protein (Fig. 4C) and mRNA (Supplementary Fig. 3A) levels. Thus, *ZBTB4* and miR-576-5p expression levels were negatively correlated.

Notably, the luciferase activity in HEK-293 T cells transfected with the wild-type *ZBTB4* 3'-UTR vector was significantly reduced when miR-576-5p was overexpressed. In contrast, the luciferase activity of those transfected with the mutant vector did not change significantly (Fig. 4F), indicating that miR-576-5p directly targeted *ZBTB4*.

### ZBTB4 inhibits the proliferation, invasion, and migration capacity of EC cells

**Fig. 5:**
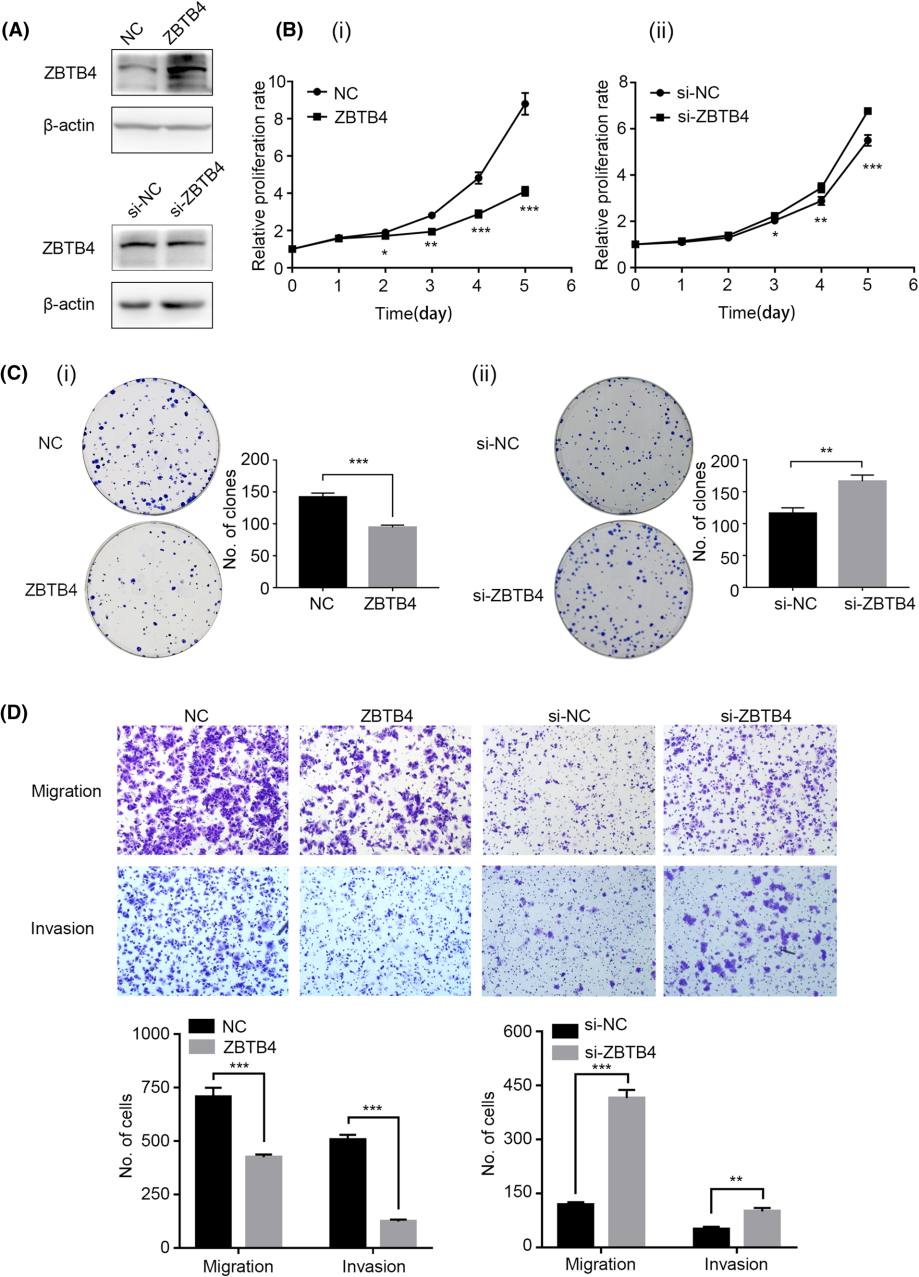
ZBTB4 inhibited proliferation and metastasis in Ishikawa cells in vitro. **A** The protein expression of ZBTB4 after overexpression and knockdown of ZBTB4. **B** the effect of ZBTB4 on the proliferation of Ishikawa cells. (i) Overexpression of ZBTB4 inhibited the proliferation of the Ishikawa cell line. (ii) Downregulation of ZBTB4 promoted Ishikawa proliferation. **C** The effect of ZBTB4 on colony formation of Ishikawa. (i) Knockdown of ZBTB4 facilitated the colony formation of Ishikawa cells. (ii) Overexpression of ZBTB4 did the opposite effect. **D** Effect of ZBTB4 on migration and invasion ability of Ishikawa cell line. *p < 0.05, **p < 0.01, ***p < 0.001

To confirm the functions of *ZBTB4* in EC cells, we downregulated or upregulated the expression of *ZBTB4* in Ishikawa cells by transfecting the cells with siRNA or pEnter-*ZBTB4*, respectively (Fig. 5A). MTT assay revealed that downregulation of *ZBTB4* prominently promoted the proliferation of Ishikawa cells, but upregulation of ZBTB4 inhibited the proliferation of these cells (Fig. 5B). Moreover, the clonogenic assays demonstrated that *ZBTB4* knockdown notably promoted the clonogenic efficiency of the cells, and overexpression of ZBTB4 inhibited the colony formation capacity (Fig. 5C). Additionally, the knockdown of *ZBTB4* promoted the metastasis capacity of Ishikawa cells, but overexpression of ZBTB4 inhibited the invasion and migration capacity of these cells (Fig. 5D).

### miR-576-5p promotes the proliferation and metastasis capacity of EC cells by directly targeting ZBTB4

**Fig. 6:**
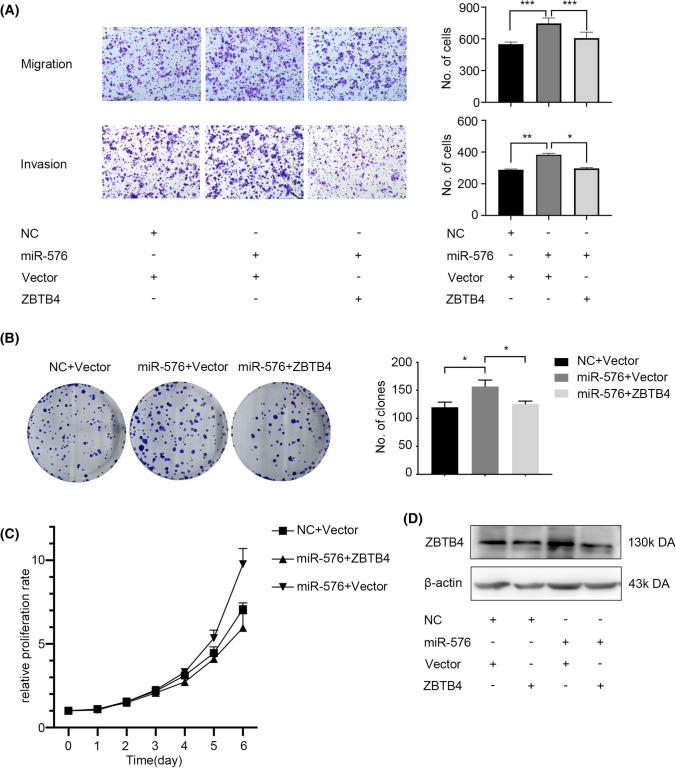
The effect of ZBTB4 on EC cell lines can be rescued by the introduction of miR-576. **A** Transwell migration and invasion assay after treatment on Ishikawa cells. **B** Colony-formation assay after treatment on Ishikawa cells. **C** MTT cell proliferation assay after treatment on Ishikawa cells. **D** The change of protein level after co-transfected miR-576 mimics and pEnter-ZBTB4. *p < 0.05, **p < 0.01, ***p < 0.001

To confirm that the carcinogenic effects of miR-576-5p in EC cells were achieved via *ZBTB4*, we performed rescue experiments. Transient co-transfection of miR-576-5p mimics and pEnter-*ZBTB4* plasmids into Ishikawa cells reversed miRNA-576-promoted cell proliferation and colony-forming ability, as revealed by MTT and clone formation assays (Fig. 6B, C). Additionally, *ZBTB4* upregulation induced by miR-576-5p could reverse the invasion and migration capacity of Ishikawa cells (Fig. 6A). Furthermore, we tested the protein expression level of *ZBTB4* after co-transfecting the cells with pEnter-*ZBTB4* plasmid and miR-576-5p mimics using Western blots. We found that the level of *ZBTB4* upregulated by pEnter-*ZBTB4* could be reversed by introducing miR-576-5p mimics (Fig. 6D). In summary, we showed that miR-576-5p could regulate the oncogenic effect of Ishikawa cells by targeting *ZBTB4*.

**Fig. 7:**
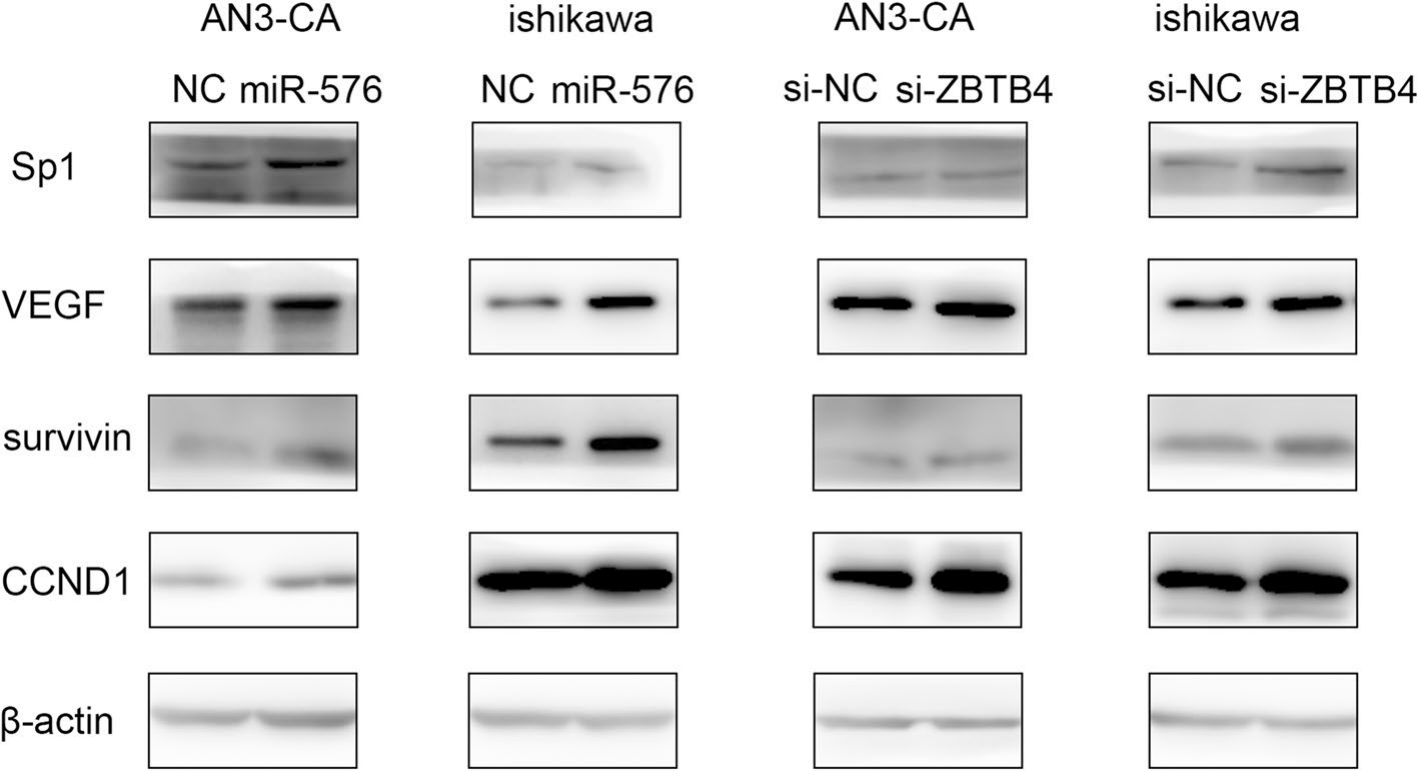
ZBTB4 inhibited EC cell line proliferation, migration, and invasion via specificity protein axis. Overexpression of miR-576 could inhibit the expression of ZBTB4 but could increase Sp1 and Sp-regulated genes (VEGF, survivin, CCND1) expression. Knockdown miR-576 did the opposite effect. The inhibition of ZBTB4 could increase the expression of Sp1 and Sp-regulated gene products

In addition, previous studies have confirmed that inhibition of *ZBTB4* could increase the expressions of *Sp1* and *Sp1*-regulated genes, such as VEGF, survivin, and CCND1 [21]. In this study, we found that upregulation of miR-576-5p could increase the expressions of Sp1 and Sp1-regulated genes, and inhibition of *ZBTB4* could achieve the same effects (Fig. 7). Several earlier studies have already confirmed that *Sp1* and *Sp1*-regulated genes were related to the development of EC [27, 28]. Thus, our results indicated that *ZBTB4* is a potential target of miR-576-5p and their interaction affects EC cell growth and metastasis.

## Discussion

In 2022, it is estimated that there will be approximately 65,950 new onsets and 12,550 deaths of uterine cancer in the United States [1]. The number of EC cases in young women has risen sharply in the last 10 years because of early-onset obesity [29]. Although the mortality rates of most other cancers decrease, the mortality rate of EC increased over the past decade [4]. In 2013, a genomic classification of EC was proposed by the Cancer Genome Atlas (TCGA) [5]. The molecular categories can distinguish groups with different prognoses, stratify risk in EC patients, and provide hints for treatment methods [30]. EC Patients in the POLE subgroup had a relatively young age of onset and significantly better clinical prognosis [31–33]. And the copy number high subgroup had the most unfavorable prognosis than the other three subgroups [33, 34]. By analyzing TCGA data, we showed that miR-576-5p was highly expressed in EC tissues, especially in the POLE subgroup. And the high miR-576 expression in the copy number- high subgroup is related to a shorter PFI time. Additionally, a high expression level of miR-576 was correlated with an earlier age of EC onset. Functional analysis indicated that upregulated miR-576-5p expression could facilitate EC cells’s migration, invasion, and proliferation.

A single miRNA is usually identified by targeting the corresponding genes with multiple functions. We found that ZBTB4 is a potential target of miR-576-5p. *ZBTB4* is a member of the *ZBTB* family, which encodes a transcriptional repressor [35]. *ZBTB4* can bind to methylated DNA, and the co-repressor complexes interact with unmethylated consensus sequences to silence genes by chromatin compaction [35]. *ZBTB4* expression level is de-regulated in many cancers, such as prostate cancer, breast cancer, and lung cancer. The low expression of *ZBTB4* is associated with the increased possibility of relapse of the cancers mentioned above [20–23]. In our study, we found that *ZBTB4* is de-regulated in EC cells, and the downregulation of *ZBTB4* is correlated with enhanced invasion and proliferation capacity of these cells. Moreover, at the cellular level, *ZBTB4* expression is negatively correlated with miR-576-5p expression.

The loss of *ZBTB4* promotes the induction of expression of P21, and cells are prone to survival rather than apoptosis after P53 activation [36]. Furthermore, the recruitment of *ZBTB4* to the *P21* promoter is mediated by the former’s association with *MIZ1 *[37]. In human cell lines, the absence of ZBTB4 causes genomic instability and mitotic abnormalities and weakens mitotic detection sites. Moreover, a lack of *ZBTB4* induces skin tumorigenesis in mice [38]. Downregulation of *ZBTB4* in ES cells increases *Ki-67* and *PCNA* expressions [23]. Previous research has shown that *ZBTB4* downregulates *Sp* gene expression and affects the expression of Sp-related oncogenes, such as survivin, VEGF, VEGFR*,* and *CCND1 *[21, 23]. Our study found that *ZBTB4* loss is associated with an increase in the expression level of *Sp1*. Further, we showed that both overexpression of *ZBTB4* and inhibition of miR-576-5p decreased the expression of *Sp1*, *VEGF*, and *survivin* in EC cells. Taken together, the miR-576-5p/*ZBTB4/Sp1* axis might work closely during EC tumorigenesis.

In summary, we revealed that miR-576-5p could affect the proliferation, migration, and invasion capacity of EC cells by by specifically targeting the ZBTB4/Sp1 axis. However, there are some limitations of this study. For instance, the relationships between miR-576-5p and EC patients in the POLE-ultramutated subgroup need to be further studies; the potential molecular mechanism underlying miR-576-5p upregulation remain to be further explored.

## Supplementary Information

Below is the link to the electronic supplementary material.

Supplementary file1 (DOCX 12 KB)

Supplementary file2 (DOCX 12 KB)
