# Supplementary material for: miRNA-576-5p promotes endometrial cancer cell growth and metastasis by targeting ZBTB4
Source: Clin Transl Oncol. 2022 Dec 20;25(3):706–20. doi: 10.1007/s12094-022-02976-8 (PMC9941281; doi:10.1007/s12094-022-02976-8)
Supplement: Supplementary file 1 — Supplementary file1 (DOCX 12 KB) [file 12094_2022_2976_MOESM1_ESM.docx]

Supplementary Table.1 Sequence of primers

| Primer Name | Primer Sequence (5’ to 3’) |
| --- | --- |
| miRNA-576(F) | ATTCTAATTTCTCCACGTCTTT |
| miRNA-576(R) | GCAGGGTCCGAGGTATTC |
| U6(F) | TGGAACGCTTCACGAATTTGCG |
| U6(R) | GGAACGATACAGAGAAGATTAGC |
| ZBTB4(F) | TCCCTTTTGCACTGAGGCTT |
| ZBTB4(R) | AGAAGG GACTTGAAGCAGCC |
| Sp1(F) | CCACCATGAGCGACCAAGAT |
| Sp1(R) | GTAGCCCCAGAGGAGGAAGA |
| ACTB(F) | CATGTACGTTGCTATCCAGGC |
| ACTB(R) | CTCCTTAATGTCACGCACGAT |
