# Supplementary material for: miRNA-576-5p promotes endometrial cancer cell growth and metastasis by targeting ZBTB4
Source: Clin Transl Oncol. 2022 Dec 20;25(3):706–20. doi: 10.1007/s12094-022-02976-8 (PMC9941281; doi:10.1007/s12094-022-02976-8)
Supplement: Supplementary file 2 — Supplementary file2 (DOCX 12 KB) [file 12094_2022_2976_MOESM2_ESM.docx]

Supplementary Table 2 Sequence of si-RNAs

| Si-RNAs | Primer Sequence (5’ to 3’) |
| --- | --- |
| miRNA inhibitor NC | CAGUACUUUUGUGUAGUACAA |
| Hsa-miR-576-5p inhibitor | AAAGACGUGGAGAAAUUAGAAU |
| Negative control | UUCUCCGAACGUGUCACGUTT  ACGUGACACGUUCGGAGAATT |
| Hsa-miR-576-5p mimics | AUUCUAAUUUCUCCACGUCUUU  AGACGUGGAGAAAUUAGAAUUU |
| ZBTB4 siRNA-1 | CGCAAAAUUUAGAAUUCUATT  UAGAAUUCUAAAUUUUGCGTT |
| ZBTB4 siRNA-2 | GAAGAGACACAGCAAUGUATT  UACAUUGCUGUGUCUCUUCTT |
